# Assessing Blood Pressure Effects of Anti-CGRP Antibody Treatment in Migraine: A Retrospective Real-World Analysis

**DOI:** 10.3390/biomedicines13051027

**Published:** 2025-04-24

**Authors:** Katharina Kaltseis, Michael Thomas Eller, Lena Gufler, Gregor Broessner, Florian Frank

**Affiliations:** Department of Neurology, Medical University of Innsbruck, 6020 Innsbruck, Austria

**Keywords:** CGRP, monoclonal antibodies, real-world data, hypertension

## Abstract

**Background:** Anti-CGRP (receptor) antibodies are approved for the preventive treatment of migraines and are increasingly used due to their favorable safety profile and potent efficacy. However, as CGRP is one of the most potent vasodilators, concerns have been raised regarding the possible impact of these drugs on arterial blood pressure. **Methods:** We present a retrospective cohort study at a tertiary headache center including a total of 259 patients with episodic and chronic migraine who received anti-CGRP antibody treatment for migraine prevention. Blood pressure was measured in a hospital using a standardized setting at baseline and at least at two follow-up visits. Significant increase in blood pressure was defined as an increase in systolic blood pressure ≥ 20 mmHg and/or an increase in diastolic blood pressure ≥ 10 mmHg from the baseline value. **Results:** Mean age of our population was 39.9 years (±12.1), and 217 (83.8%) were female. Blood pressure measurements between T0 and T2, incorporating all CGRP-antibody groups, showed a significant reduction in systolic (−3.3 mmHg; *p* = 0.001) and diastolic blood pressure (−2.3 mmHg; *p* = 0.021), respectively. The repeated-measures generalized linear model analysis revealed no significant variations between the CGRP antibodies relative to blood pressure. The most robust factor predicting systolic hypertensive measurements in the course of anti-CGRP antibody treatment was pre-existing hypertension at baseline (sum of mean squares 7.4; *p* = 0.019). **Conclusions:** Our data indicate that treatment with anti-CGRP (receptor) antibodies does not significantly increase blood pressure. However, it seems to be important to monitor patients with pre-existing arterial hypertension.

## 1. Introduction

The discovery of the neuropeptide calcitonin gene-related peptide (CGRP) and the understanding of its key role in the pathophysiology of migraine gave new momentum to the treatment options for this primary headache disorder [1,2,3].

Currently, there are four monoclonal antibodies available: one targeting the CGRP receptor (erenumab) and three targeting the CGRP ligand (eptinezumab, fremanezumab, galcanezumab).

So far, real-world data seem to confirm the favorable side effect profile of anti-CGRP (receptor) antibodies [4,5]. However, concerns have been expressed regarding the possible impact on arterial blood pressure [6]. Given the fact that CGRP is one of the most potent vasodilators, based on its presumable beneficial effect in hypertension and the associated higher risks of cardiovascular events in migraine patients, it is crucial to ensure the safe usage of these substances in this patient population without the risk of developing hypertension.

Two meta-analyses of Phase III studies on galcanezumab [7] as well as erenumab [8] did not reveal an increased risk of strokes, ischemic heart disease, or elevated blood pressure. However, it is important to note that a post-marketing follow-up analysis [9] and recently published studies from the Netherlands [10] and the United States [11] present a different perspective on increased blood pressure during treatment with erenumab (see Table 1). In the former, elevated blood pressure values were observed within the first week of treatment with erenumab; in the latter, the authors found that the blood pressure was significantly increased after initiation of an anti-CGRP (receptor) antibody, ultimately leading to the necessity of antihypertensive treatment in some patients. Based on this limited evidence, it can be concluded that further research is needed to fully understand the potential risks associated with this treatment.

As these drugs are increasingly being used for migraine prevention, an increase in blood pressure could have far-reaching consequences and could require potentially intensive monitoring of the patients.

In the present study, we hypothesized that preventative treatment with an anti-CGRP (receptor) antibody in migraine patients has no effect on blood pressure.

## 2. Materials and Methods

### 2.1. Study Design and Participants

This analysis is a secondary analysis of previously collected data. We performed a post-hoc analysis at the tertiary headache outpatient clinic of the Medical University of Innsbruck using the electronic medical outpatient records.

Therefore, we included all female and male patients aged 18 to 70 years who were diagnosed by a headache specialist with episodic or chronic migraine with or without aura according to the ICHD-III diagnostic criteria [12]. All patients fulfilled the European Medicines Agency (EMA) and local criteria for initiation an anti-CGRP (receptor) antibody as prophylactic treatment between the 1 January 2018 and 31 May 2023. As eptinezumab has only been approved in Austria since January 2023, patients initially received erenumab, galcanezumab, or fremanezumab as their first monoclonal antibody. We also included patients who switched from one antibody to another either due to side effects or loss/lack of efficacy. Women of child-bearing potential were advised to use basic methods of contraception.

**Figure 1:**
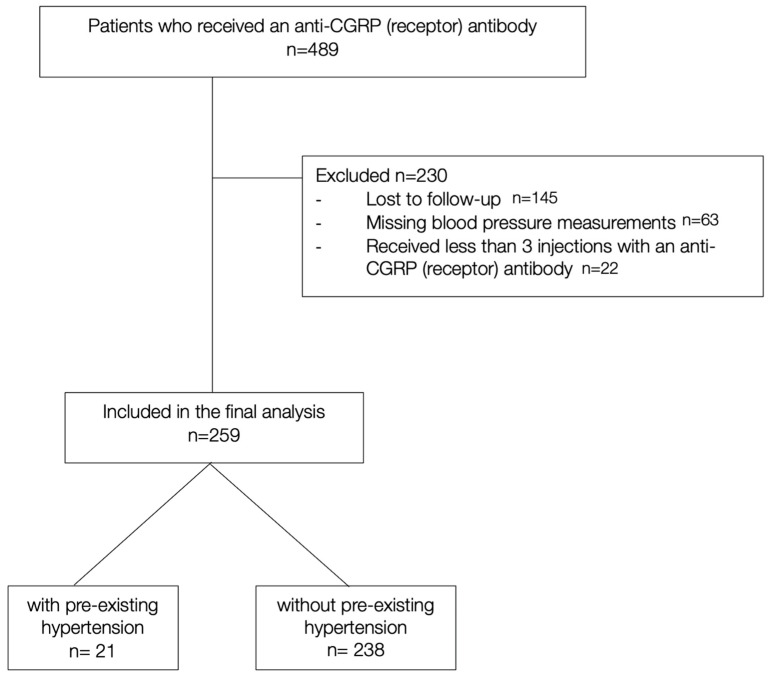
Flow chart of patient selection.

Patients were included in the final analysis if (i) they received at least three injections with any anti-CGRP (receptor) antibody and (ii) if there were at least two blood pressure measurements available after treatment initiation (see Figure 1).

None of the patients in our study were prescribed gepants, neither as abortive nor as preventive treatment.

History of uncontrolled hypertension at baseline was considered as exclusion criteria for the initiation of any CGRP-targeting therapies.

This study was conducted according to the declaration of Helsinki and was approved by the local ethics committee [ECS 1208/2023]. Written informed consent was waived based on the nature of the study and in accordance with our local ethics committee.

### 2.2. Blood Pressure Measurements

As part of the clinical routine, vital signs including systolic and diastolic blood pressure as well as heart rate were routinely collected from the migraine patients during each visit to the outpatient department. According to the up-to-date guidelines of the European Society of Cardiology (ESC) and the European Society of Hypertension (ESH), blood pressure and heart rate were measured with an automated validated device with an appropriately sized cuff after the patient had been sitting for five minutes with both feet on the ground [13].

**Figure 2:**
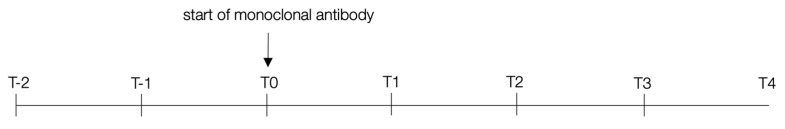
Individual time points of routine blood pressure measurement before (T-2, T-1 and T0) and after (T1–T4) the initiation of an anti-CGRP (receptor) antibody in migraine patients. Follow-up assessments were conducted over a period of 3 to 6 months.

A schematic presentation of the timepoints of the blood pressure measurements is given in Figure 2.

The time between the follow-up visits at our outpatient department and, therefore, the individual blood pressure measurements varied between 3 and 6 months.

In line with previous published studies on this topic, a significant increase in blood pressure was defined as an increase in systolic blood pressure ≥ 20 mmHg and/or an increase in diastolic blood pressure ≥ 10 mmHg from the average baseline value, i.e., before the start of the anti-CGRP (receptor) antibody [10].

Pre-existing arterial hypertension, diagnosed by a general practitioner or a specialist, and blood pressure medication (beta-blocker, angiotensin-converting enzyme inhibitor, calcium channel blocker and angiotensin II receptor blocker) were extracted from the electronic patient records.

### 2.3. Statistical Analysis

Baseline characteristics such as age, sex, migraine diagnosis as well as proportion of patients with headache-free days, medication overuse headache, arterial hypertension and concomitant antihypertensive medication were presented as means and standard deviation or ranges according to the distribution. Clinical variables were also presented as means with standard deviation. Normality of distribution of the variables was evaluated with Kolmogorov–Smirnov and Shapiro–Wilk tests. Group differences in the baseline characteristics, as well as clinical variables between each antibody therapy at T0 were assessed using ANOVA and a Kruskal–Wallis test.

A repeated-measures generalized linear model analysis to test for the influence of antibody prescription on vital parameters was fitted with age, aura, and antihypertensive medication as covariates and computed for blood pressure and pulse with each antibody as a between-subjects factor.

## 3. Results

A total of 259 patients received a monoclonal CGRP-^®^ antibody and were included in our final analysis set. At baseline, 116 (44.8%) fulfilled the criteria for chronic migraine, and 67 (25.9%) were classified with migraine with aura. Among all included patients, 33 (12.7%) reported no headache-free interval at baseline and 66 (25.5%) had comorbid medication-overuse headache (MOH).

Most patients initially received erenumab (70 mg or 140 mg, administered every 4 weeks subcutaneously) as the first monoclonal antibody (n = 128, 49.4%), and fremanezumab (225 mg monthly or 675 mg quarterly, administered subcutaneously) was initially started in 74 patients (28.6%), galcanezumab (120 mg monthly with a loading dose of 240 mg, administered subcutaneously) in 56 patients (21.6%). Eptinezumab (100 mg, administered quarterly via intravenous infusion) was the initial choice in only one patient (0.4%).

When an antibody switch was attempted (n = 46, 17.7%), galcanezumab was the second antibody in half of the cases (n = 23), followed by erenumab (n = 12, 26.1%) and fremanezumab (n = 11, 23.9%). Only a few patients were deemed to be candidates for a third switch (n = 15, 5.8%) (fremanezumab n = 9, 60.0%; galcanezumab n = 4, 26.7%, eptinezumab n = 2, 13.3%), and two patients were ultimately switched a fourth time to eptinezumab.

Mean age of our population was 39.9 years (±12.1), and 217 (83.8%) were female. The baseline characteristics (see Table 2) did not differ statistically between subcutaneously applied antibodies. The eptinezumab group was underpowered for a conclusive statistical analysis.

Twenty-one patients (8.1%) had a known arterial hypertension at baseline, and thirteen (5.0%) were on active antihypertensive medication prior to the initiation of anti-CGRP receptor antibodies. If on antihypertensives, patients remained on a stable dose during the duration of the anti-CGRP (receptor) antibody treatment.

At baseline, 64 patients (24.7%) presented with an arterial hypertensive systolic measurement > 140 mmHg, and 30 (11.5%) had an elevated baseline diastolic blood pressure (>90 mmHg). These patients were advised to regularly monitor their blood pressure at home, record their readings, and present to their general practitioner. None of these patients required the initiation of antihypertensive treatment. Most data were available for the repetitive measurements between T0 (baseline) and T2 (Table 3).

The median duration between T0 and T2 was 7 months. Blood pressure measurements between T0 and T2, incorporating all CGRP-antibody groups, showed significant results.

**Figure 3:**
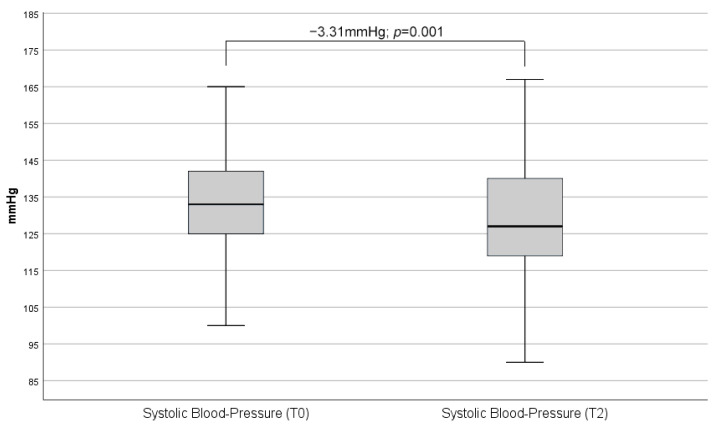
Comparison of overall mean systolic blood pressure measurements between baseline (T0) and second on-site visit (T2). In the combined analysis of all administered CGRP-antibody therapies, a mean decrease in systolic blood pressure of 3.3 mmHg was observed. The boxes represent the interquartile range with median and error bars represent range excluding outliers.

**Figure 4:**
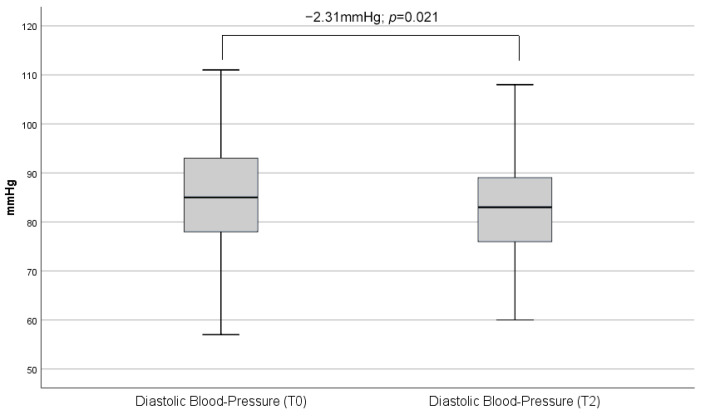
Comparison of overall mean diastolic blood pressure measurements between baseline (T0) and second on-site visit (T2). In the combined analysis of all administered CGRP antibody therapies, a mean decrease in systolic blood pressure of 2.3 mmHg was observed. The boxes represent the interquartile range with median and error bars represent range excluding outliers.

As shown below, there was a reduction in both systolic (−3.3 mmHg; *p* = 0.001) as well as diastolic (−2.3 mmHg; *p* = 0.021) blood pressure (Figure 3 and Figure 4).

The repeated-measures generalized linear model analysis revealed no significant variations between the CGRP antibodies relative to blood pressure. We did not find any statistically significant correlation and/or difference between subjects of either drug group, age and gender.

The most robust factor predicting systolic hypertensive measurements in the course of our study, as analyzed with our model, was a known arterial hypertension at baseline (*p* = 0.019). No factor was significantly influencing diastolic measurements or heart rate.

## 4. Discussion

In our retrospective data analysis comprising a total of 259 patients, we were able to demonstrate that prophylactic treatment with a monoclonal antibody in migraine patients did not significantly increase arterial blood pressure during the follow up.

In the scope of our analysis, we present data for three currently available monoclonal antibodies—erenumab, fremanezumab and galcanezumab—which are approved for the prophylactic treatment of migraine attacks. A conclusive analysis of eptinezumab was not possible due to the small sample size. Most patients initially received erenumab as their first monoclonal antibody, as it was the first one to be approved in Europe. In contrast to a previous study [10], we could not observe any difference in the effect on the blood pressure neither by nor between the individual monoclonal antibodies. We even included patients who were switched to a second, third. or even fourth antibody, again, without any impact on blood pressure.

The most robust factor predicting systolic hypertensive measurements during our study was pre-existing arterial hypertension, which was the case for 21 (8.1%) of our patients. Similarly, two studies reported an exacerbation of pre-existing hypertension during anti-CGRP (receptor) treatment [14,15].

Therefore, special attention should be given to these patients including regular repetitive pressure monitoring and early initiation of antihypertensive therapy, if indicated.

The cut-off values used in this study for diagnosing arterial hypertension align with the current guidelines of the ESC and the ESH [13]. These guidelines indicate that systolic blood pressure values (measured at a doctor’s office) above 140 mmHg and diastolic values above 90 mmHg are considered as hypertension.

To minimize the effect of a possible ‘white coat hypertension’, our patients had their blood pressure measured in standardized settings at baseline—thus prior to the start of the antibody therapy—and during at least two different follow-up appointments.

A limitation of our study is its retrospective nature. Hence, in order to ensure high data quality, we had to exclude some participants due to missing data or loss to follow-up. However, we firmly believe that our study sample consisting of 83.8% (n = 217) female participants with a mean age of 39.9 ± 12.1 years is representative of the general migraine population. Another important limitation of our study is the absence of a control group; therefore, we cannot rule out the influence of confounding variables, natural disease progression, or external factors that may have affected the observed outcomes.

In recent years, numerous risk factors for cerebrovascular diseases were identified, among them obesity, hypercholesterinemia, diabetes, and smoking. Hypertension, however, is known as the most prevalent [16] and presumably also as the most relevant controllable risk factor for stroke.

Several studies could show that the relative risk of an ischemic stroke in migraine patients with aura ranges from 2.14 to 2.41 [17,18]; especially women younger than 45 years who use an oral hormonal contraceptive and smoke are at risk [19]. Active migraine, i.e., migraine attacks in the last 12 months, as well as high attack frequency further increase the risk for a cerebrovascular event [17].

Hence, it is of utmost interest to the headache community to provide this vulnerable patient group with a treatment that is both effective and safe.

## 5. Conclusions

Our extensive retrospective analysis strongly supports the vascular safety of monoclonal antibodies that interfere with the CGRP pathway in migraine patients. This is consistent with data from approval studies and long-term observational studies. While it is important to consider the potential inhibitory effect on vasodilation of this drug class, it is worth noting that there are numerous redundant endothelial effectors that counteract the antagonism of CGRP, at least in healthy patients. Overall, our findings suggest that the benefits of these monoclonal antibodies outweigh any potential risks. In clinical practice, it is recommended to proactively address arterial hypertension, a known cardiovascular risk factor, in patients with migraine, based on our data.

## Figures and Tables

**Table 1:** Overview of study characteristics and key findings on CGRP antibodies and hypertension.

Study (Ref)	Oakes et al., 2019 [7]	Dodick et al., 2021 [8]	Saelyl et al., 2020 [9]	De Vries Lentsch et al., 2022 [10]	Chhabra et al., 2024 [11]
Cases (n)	2886	2443	61	196	335
Population	EM, CM	EM, CM	EM, CM	EM, CM	EM, CM
Study design	Pooled safety analysis of three Phase 3, double-blind, placebo-controlled studies	Pooled analysis of four Phase 2 and 3 double blind, placebo-controlled clinical trials	Retrospective, postmarketing FDA-report analysis (Cave: exclusion of 222 reports) ^2^	Prospective 1-year follow-up study	Observational retrospective cohort study
Treatment (n)	Galcanezumab 120 mg/month (705); Galcanezumab 240 mg/month (730); Placebo (1451)	Erenumab 70 mg/month (893); Erenumab 140 mg/month (507); Placebo (1043)	Erenumab (any dosing regime)	Erenumab (any dosing regime; 109); Fremanezumab 225 mg/month (87); Control group (109)	Erenumab 70 mg/month (190); Erenumab 140 mg (144); 1 Missing
Duration	2 × 6 months (EM) and 1 × 3 months (CM)	12 weeks (pooled)	^3^ n.a.	6–12 months (Cave: n = 96 at 12 months)	^6^ 20.5 (13.3–35.3) weeks
Hypertension at baseline; n (%)	135 (9.3) Placebo group 103 (7.2) Treatment group	93 (8.9) Placebo group 85 (6.1) Treatment group	19 (31.1) Treatment group	0 (0.0) Control group 7 (3.6) Treatment group	70 (20.9) Treatment group
Worsening of blood pressure under treatment; n (%)	^1^ 141(10.0) Placebo group 156 (11.0) Treatment group	9 (0.9) Placebo 8 (0.6) Treatment group	^4^ 27 (44.2) Treatment group	0 (0.0) Control group ^5^ 76 (38.8%) Treatment group	78 (23.3) Treatment group
Initiation/Increase of antihypertensive medication under treatment; n(%)	34 (2.3) Placebo group 23 (1.6) Treatment group	12 (1.2) Placebo group 8 (0.6) Treatment group	25 (41.0) Treatment group	0 (0.0) Control group 4 (2.0) Treatment group	23 (6.8%) Treatment group

EM: episodic migraine; CM: chronic migraine; BP: blood pressure ^1^ Defined as increase to ≥140 mmHg systolic blood pressure or 20 mmHg increase from baseline, and/or ≥90 mmHg diastolic blood pressure or increase of ≥10 mmHg, including patients with and without baseline hypertension; no differentiation possible between isolated BP increase or combined (systolic, diastolic) increase, Cave: 1:1:2 randomization; ^2^ FAERS database search (FDA adverse event reporting system) evaluating significant BP (blood pressure) elevation, strong alternative causes for BP elevation excluded; ^3^ n.a. = not available; ^4^ initiation of a pharmacological intervention OR emergency department visit/hospitalization for clinically emergent de novo or worsening of controlled preexisting hypertension; ^5^ systolic BP rise of ≥20 mm Hg and/or a diastolic BP rise ≥ 10 mm Hg at any time during the course of treatment; ^6^ values are given as median and interquartile range (IQR).

**Table 2:** Baseline characteristics at T0.

	Total (n = 259)	Erenumab (n = 128)	Galcanezumab (n = 56)	Fremanezumab (n = 74)	Eptinezumab (n = 1)
Female, n (%)	217 (83.8)	103 (80.5)	49 (87.5)	63 (85.1)	1 (100.0)
Age, y, mean ± SD (range)	39.9 ± 12.1 (18–74)	40.5 ± 12.0 (18–74)	38.9 ± 11.6 (18–61)	39.5 ± 12.8 (18–66)	50
Aura, n (%)	67 (25.9)	31 (24.2)	15 (26.8)	21 (28.4)	0 (0.0)
Chronic migraine, n (%)	116 (44.8)	54 (42.2)	29 (51.8)	33 (44.6)	0 (0.0)
MOH, n (%)	66 (25.5)	30 (23.4)	15 (26.8)	20 (27.0)	0 (0.0)
HFD, n (%)	226 (87.3)	110 (85.9)	47 (83.9)	67 (90.5)	1 (100.0)
Hypertension, n (%)	21 (8.1)	8 (6.3)	5 (8.9)	8 (10.8)	0 (0.0)
Prior blood pressure medication, n (%)	13 (5.0)	7 (5.5)	2 (3.6)	4 (5.4)	0 (0.0)
Systolic BP, mean ± SD	134.3 ± 14.9	133.4 ± 14.7	137.0 ± 15.1	133.9 ± 15.2	138.0
Diastolic BP, mean ± SD	85.5 ± 11.6	85.2 ± 8.9	56.7 ± 15.6	85.3 ± 12.3	78.0
Heart Rate, mean ± SD	83.3 ±15.7	82.4 ± 16.5	85.4 ± 16.3	83.4 ± 14.1	76.0

BP: blood pressure; MOH: medication-overuse headache; HFD: patients reporting headache-free days; SD: standard deviation.

**Table 3:** Descriptive presentation of the measurements at time points T0 and T2.

	T0 (n = 220)	T2 (n = 122)
Systolic BP, mean ± SD	134.3 ± 14.9	129.5 ± 15.9
Diastolic BP, mean ± SD	85.5 ± 11.6	83.3 ± 15.7
Heart Rate, mean ± SD	83.3 ± 15.7	79.6 ± 13.3

BP: blood pressure; SD: standard deviation.

## Data Availability

The original contributions presented in this study are included in the article. Further inquiries can be directed to the corresponding author.
